# Perceptions of Nigerian medical students regarding their preparedness for precision medicine: a cross-sectional survey in Lagos, Nigeria

**DOI:** 10.1186/s12909-023-04841-w

**Published:** 2023-11-17

**Authors:** Chibuzor F. Ogamba, Alero A. Roberts, Sharon C. Ajudua, Mosopefoluwa O. Akinwale, Fuhad M. Jeje, Festus O. Ibe, Moses M. Afolayan, Yetunde A. Kuyinu

**Affiliations:** 1 Nuffield Department of Population Health, University of Oxford, Oxford, UK; 2 Department of Community Health and Primary Care, College of Medicine, University of Lagos, Lagos, Nigeria; 3 Faculty of Clinical Sciences, College of Medicine, University of Lagos, Lagos, Nigeria; 4 Faculty of Clinical Sciences, Lagos State University College of Medicine, Lagos, Nigeria; 5 Department of Medicine, Albert Einstein Medical Center Philadelphia, Philadelphia, PA USA; 6 Department of Community Health and Primary Health Care, Lagos State University College of Medicine, Lagos, Nigeria

**Keywords:** Precision medicine, Medical genomics, Medical education, Nigeria.

## Abstract

**Background:**

Advances in precision medicine in Nigeria suggest improving genomics education and competency among healthcare practitioners to facilitate clinical translation. Due to the scarcity of research in this area, this study aimed to assess Nigerian medical students’ perceptions about their preparedness to integrate precision medicine into their future clinical practice.

**Methods:**

This was an institution-based cross-sectional study of medicine and surgery students in their clinical years attending the two fully accredited colleges of medicine in Lagos, Nigeria, between April and October 2022 using an adapted tool administered via Google Forms. The survey assessed their awareness, perceptions about knowledge, ability, and attitudes toward precision medicine, ethical concerns, and perceptions about their education in precision medicine. Multivariate linear regression models were used to assess factors associated with students’ perceptions of their knowledge, ability, and attitudes.

**Results:**

A total of 300 students completed the questionnaires with a response rate of 40%. Awareness of genomic medicine terminology was high (92.0%). Responses to knowledge and ability questions revealed notable gaps, however, respondents had positive attitude scores overall. Higher medical school year was independently associated with lower knowledge (p_trend_ = 0.003) and ability (p_trend_ = 0.005) scores, and knowledge score was independently associated with a higher ability score (β: 0.76 95%CI: 0.67, 0.84; p < 0.001). Attitude scores significantly increased with increasing medical school year (p_trend_ = 0.04). The respondents mostly indicated concerns about government and corporate bodies’ misuse of genomic data (35.7%) and the widening of socioeconomic disparities (34.0%). Although 65.0% of the respondents thought it important to learn about precision medicine, only 11.3% felt that their education had adequately prepared them for precision medicine, knew who to ask questions regarding genomic testing (10.7%), and felt their professors had encouraged the use of precision medicine (10.3%).

**Conclusion:**

Despite high awareness of precision medicine terminology and overall positive attitudes, our findings highlight gaps in knowledge and ability to integrate genomics into the care of patients and a need to improve precision medicine education among Nigerian medical students.

**Supplementary Information:**

The online version contains supplementary material available at 10.1186/s12909-023-04841-w.

## Introduction

Precision medicine involves using individualized patient or population data, especially genomic data and other omics data, lifestyle, and environmental factors, to tailor approaches to disease prevention, diagnosis, and treatment [1–3]. Genomic medicine, which utilizes genomic data in disease prevention and management, has had varied applications in healthcare, including prenatal and newborn genetic screening, cancer screening and therapy, pharmacogenomics and pathogen genome sequencing, to mention a few [4].

Unfortunately, despite the revolutionary advancements in medical genomics, these advances have been restricted mainly to developed countries, as was earlier feared [5]. Due to the under-representation of African countries in particular in these efforts, coupled with poorer resources and limitations in infrastructure on the continent, the available knowledge and evidence for the implementation of precision medicine approaches lag behind those of the developed world and global challenges to precision medicine are faced on a uniquely larger scale in countries like in sub-Saharan Africa [4, 6]. Being the most populous country in Africa, Nigeria is experiencing a rapidly changing genomic medicine landscape due to recent international collaborations and funding efforts geared toward research and training [7–9]. Regardless, the current situation in Nigeria is still far from adequate, with significant unmet needs [7, 10].

One of the frameworks for addressing the need for developing precision medicine in Africa is improving the training of physicians in genomic medicine and its applications to the clinical management of patients [4]. With the ongoing advances in genomic medicine in Nigeria and Africa, there is a need for physicians and those in training to acquire some form of genomic literacy [4, 11]. Previous surveys among healthcare providers in other countries have revealed gaps in pre-requisite knowledge [12–15]. Many felt inadequately prepared and believed they should have more extensive exposure to genomic medicine during their training [12, 16–22].

However, to our knowledge, no study has been done in Nigeria to assess medical students’ preparedness for an era of precision medicine. A recent study among final year nursing students in Nigeria found high rates of poor knowledge and readiness to integrate genomic concepts in nursing practice [23]. With approaches to bridge the gaps in precision medicine in healthcare in Africa, this begs for undergraduate medical students to have a solid foundation to apply genomics medicine to the various specialties by equipping them with the requisite skills and knowledge needed to easily integrate them in their clinical practice, as has been underscored in other climes [12, 24–27].

Apart from expertise, different ethical, legal, and social issues (ELSI) arise in precision medicine, such as privacy and confidentiality, racial/ethnic discrimination, influence on health inequalities, and access and utilization of population genomic data by governments and corporate bodies [1]. It is necessary to understand the current perceptions of medical students in low-resource settings like Nigeria about these issues and identify training needs, knowing they are future physicians trained to practice in more global settings [1, 5].

Therefore, this study aimed to assess the preparedness of Nigerian medical students for precision medicine by assessing their awareness, perceptions regarding their knowledge and ability to adopt precision medicine approaches in the clinical management of patients, attitudes towards precision medicine as well as perceptions of ethical concerns and their education in precision medicine among medicine and surgery students in clinical years of medical school who are in the later years of their medical training.

## Methodology

### Study setting

Lagos state is located in the southwestern part of Nigeria and is the most populous state in the country [28]. This study was conducted at the College of Medicine of the University of Lagos and the Lagos State University College of Medicine, the only two fully accredited colleges of medicine in the state [29]. As of the time of this survey, Medicine and Surgery students at the University of Lagos complete basic medical sciences including anatomy, physiology, biochemistry, pathology, and pharmacology during the first three years of their training, followed by clinical rotations and specialty lectures during the next three years. At the Lagos State University, students maintain a similar curriculum but differ in starting clinical rotations in the third year while continuing pathology and pharmacology into the fourth year of their training. Basic genomic medicine concepts and terminologies are typically taught in the basic medical sciences with possible continued exposure to clinical applications in their clinical years.

### Study design

This was a cross-sectional study to assess medical students’ awareness of precision medicine terminologies, perceptions regarding their knowledge of genomic medicine concepts and their ability to apply genomic data to clinical care of patients, attitudes towards precision medicine, perceptions about ethical concerns related to precision medicine as well as about their education in precision medicine. 

### Study sample

This study was conducted among clinical students (4th – 6th year) attending both colleges of medicine and spanned between April and October 2022. The decision to focus on these three levels was to also assess the perceived effect of a continuum of exposure to genomic medicine concepts from the basic medical sciences to their clinical years. All clinical students in their 4th to 6th years of study in the Medicine and Surgery department of the University of Lagos and the Lagos State University College of Medicine were eligible to participate in the study and all those who consented to participate were included. Students not meeting the above criteria including those on transfer from medical schools outside Nigeria who may have had exposure to precision medicine were excluded from participation.

A minimum sample size of 254 was determined using the modified Cochran formula for sample size calculation in smaller populations using the estimated total number of medical students by the Medical and Dental Council of Nigeria (MDCN) quota for each college (150 for the University of Lagos, and 100 for the Lagos State University) multiplied by three (i.e. 450 and 300 students respectively) for the three clinical years [29]. The minimum sample size was increased by 10% to account for contingencies such as non-response.

The minimum number of respondents per college was proportionately determined based on each college’s MDCN quota. Convenience sampling was then used to recruit clinical students in their fourth to sixth years of medical school and recruitment continued until the survey had at least surpassed the minimum sample size. Questionnaires were disseminated through social media platforms such as online class groups and direct messages.

### Data collection tools and techniques

A self-administered structured Google Form questionnaire was adapted from previous studies. Questions on perceptions about their knowledge and ability, attitudes and education about precision medicine were adapted from Eden et al. (2016) while questions about ethical concerns were adapted from Siamoglou et al. (2021) [12, 14]. Consultant physicians in genomic medicine (international) and public health (local) assessed the face validity of the survey tool. The questionnaire consisted of open-ended, closed-ended and Likert-scaled questions and was divided into seven sections (A-G) as follows:

Section A: This section assessed the sociodemographic characteristics of the respondents, including age (in years), gender (male or female), ethnicity (Yoruba, Igbo, Others), medical school year (4th, 5th or 6th year) and interest in a career involving research (Yes or No).

Section B assessed respondents’ awareness of the terms ‘Precision Medicine’, ‘Genomic Medicine’, ‘Pharmacogenomics’, ‘Next Generation Sequencing’, and ‘Genome-guided prescribing’. Respondents were also asked to indicate their primary source of knowledge on these terminologies if they had heard of any, among options of healthcare providers, lectures, internet, media and peers.

Subsequently, a brief description of these terminologies was provided to enable respondents to continue with the rest of the questionnaire.

Section C consisted of four items assessing respondents’ perceptions regarding their knowledge of genomic testing concepts. The items were rated on a 5-point Likert scale from ‘not comfortable at all’ with their knowledge to ‘very comfortable’.

Section D consisted of four items assessing the perceptions regarding their ability to apply genomics to clinical care rated on the same 5-point Likert scale.

Section E: This section assessed respondents attitudes using two of the three subscales of the Evidence-based Practice Attitude Scale Adapting Genome-informed Interventions (EBPAS-GII) [12, 16], assessing openness to new practices (four items) and divergence of usual practice with research-based/academically developed genome-informed interventions (four items), the latter of which was reverse-scored. This section was measured on a five-point Likert scale of agreement ranging from ‘not at all’ to ‘to a very great extent’.

Section F: This section consisted of six items assessing respondents’ perception towards ethical considerations related to precision medicine rated on a five-point Likert scale from ‘not at all’ to ‘to a very great extent’.

Section G: This section consisted of four items assessing whether respondents felt their medical school curriculum had prepared them to practice precision medicine, whether their professors have encouraged the use of precision medicine, whether they know whom to ask questions regarding genomic testing, and whether they think it is important to learn about genomic medicine. These were also rated on a five-point Likert scale from ‘not at all’ to ‘to a very great extent’.

### Data analysis

Collated data were analyzed using Stata/MP-18.0. Scores were assigned to individual scaled responses and summed to derive composite knowledge, ability and attitude scores. Histograms revealed non-normal distributions in age, knowledge, ability and attitude scores. Descriptive statistics, including frequencies, medians and interquartile ranges, were used to present the distributions of the variables. The primary outcome variables were the knowledge, ability, and attitude scores, while secondary outcome variables were respondents’ perceptions about ethical concerns related to precision medicine and their education about precision medicine.

Univariate linear regression models were used to estimate the change in continuous knowledge, ability and attitude scores with age fitted as a continuous variable, and gender, ethnicity, medical school year and interest in a career involving research all fitted as categorical variables. In addition, the effect of knowledge score on change in ability and attitude scores was also examined in univariate models. Subsequently, multivariate linear regression models for each summary score were built with sequential adjustments for age, gender, medical school year, and interest in a career involving research, with additional adjustments for knowledge score as a continuous covariate in the models of ability and attitude scores. The p-value from the F-test was used to estimate the overall significance of each sequentially adjusted covariate and test for trend in all models. The likelihood ratio chi-square test was used to assess for any evidence of statistical interaction between knowledge score and medical school year in the model for attitude scores. Each model was subsequently evaluated to exclude violations of assumptions of the linear model.

Results of Likert-scaled responses were collapsed into three possible answers representing positive, neutral and negative responses for the various domains. These were presented using bar charts constructed using Microsoft Excel (2016). P-value < 0.05 was considered statistically significant.

The study findings were reported according to the Strengthening the Reporting of Observational Studies in Epidemiology (STROBE) guidelines [30]. Table S1.

### Ethics

Approval for this study was obtained from the Health Research and Ethics Committees of the Lagos State University Teaching Hospital, Ikeja, Lagos (LREC/06/10/1885) and the Lagos University Teaching Hospital Idi-araba, Lagos, Nigeria (ADM/DSCST/HREC/APP/5052). Participation in the study was entirely voluntary and respondents were required to indicate consent to participate in the study before completing the questionnaire. Respondents were guaranteed the confidentiality of their information, and no personal identification data was sought for the study. Respondents were reminded of how honest answers were required for the study.

## Results

A total of 300 medicine and surgery clinical students completed the survey (170 from the University of Lagos and 130 from Lagos State University) resulting in a 40% response rate (calculated as the number of completed questionnaires divided by the potential number of eligible participants based on the MDCN quota for both colleges). The sociodemographic characteristics of the respondents by knowledge, ability and summary scores are shown in Table 1. Respondents were 19 to 39 years old with a median age of 23 (IQR: 22–24) and slightly higher females (52.3%). At least a quarter of the respondents were from each level, with the majority from sixth (38.3%) and fifth years (36.3%). Most respondents (63.3%) indicated an interest in a career involving research.

**Table 1:** Baseline characteristics of study participants by knowledge, ability and attitude scores (N = 300)

	Total (N)	Knowledge Score	p-value	Ability Score	p-value	Attitude Score	p-value
	300	**median (IQR)**		**median (IQR)**		**median (IQR)**	
		12.00 (8.00-14.50)		11.00 (7.00–15.00)		28.00 (24.00–33.00)	
**Age (years)**							
**19–23**	161	11.00 (8.00–15.00)	0.87	11.00 (7.00–15.00)	0.79	29.00 (25.00–33.00)	0.22
**24–39**	139	12.00 (8.00–14.00)		11.00 (7.00–14.00)		28.00 (24.00–32.00)	
**Gender**							
**Female**	157	11.00 (7.00–14.00)	0.26	11.00 (7.00–14.00)	0.83	29.00 (25.00–33.00)	0.10
**Male**	143	12.00 (8.00–16.00)		12.00 (7.00–16.00)		28.00 (24.00–32.00)	
**Ethnicity**							
**Yoruba**	217	11.00 (8.00–15.00)	0.95	11.00 (7.00–15.00)	0.97	28.00 (24.00–32.00)	0.19
**Igbo**	57	11.00 (8.00–14.00)		11.00 (8.00–14.00)		29.00 (25.00–33.00)	
**Other**	26	12.00 (7.00–14.00)		12.00 (7.00–14.00)		29.00 (27.00–34.00)	
**Medical school** **year**							
**4th year**	76	12.00 (8.00-15.50)	0.036*	12.00 (8.50–16.00)	0.001**	27.00 (24.50–31.00)	0.23
**5th year**	109	12.00 (8.00–15.00)		11.00 (7.00–15.00)		29.00 (24.00–33.00)	
**6th year**	115	9.00 (6.00–13.00)		9.00 (5.00–13.00)		29.00 (24.00–33.00)	
**Interested in a career** **involving research**							
**No**	110	10.00 (7.00–13.00)	0.048*	10.00 (8.00–14.00)	0.24	28.00 (24.00–32.00)	0.12
**Yes**	190	12.00 (8.00–15.00)		12.00 (7.00–16.00)		29.00 (25.00–33.00)	

IQR: Interquartile range, *p < 0.05, **p < 0.01, p-values are from the Wilcoxon rank-sum test for two-level variables and the Kruskal-Wallis H test for > 2 levels

### Awareness of precision medicine terminologies

Most respondents (92.0%, n = 276) indicated they had heard of at least one of the precision medicine terminologies. The most commonly indicated terminology were ‘Pharmacogenomics’ (71.0%, n = 213) and ‘Genomic Medicine’ (47.7%, n = 143), while the least indicated terminologies were ‘Genome-guided prescribing’ (19.7%, n = 59) and ‘Next Generation Sequencing’ (18.0%, n = 54). Among those who had indicated awareness, the most commonly cited source of knowledge was ‘Lectures’ (49.6%, n = 137), ‘Media’ (34.4%, n = 95) and less commonly ‘Healthcare providers’ (10.1%, n = 28) and ‘Peers’ (5.1%, n = 14).

### Participants’ perception about their knowledge of genomic testing concepts

**Fig. 1:**
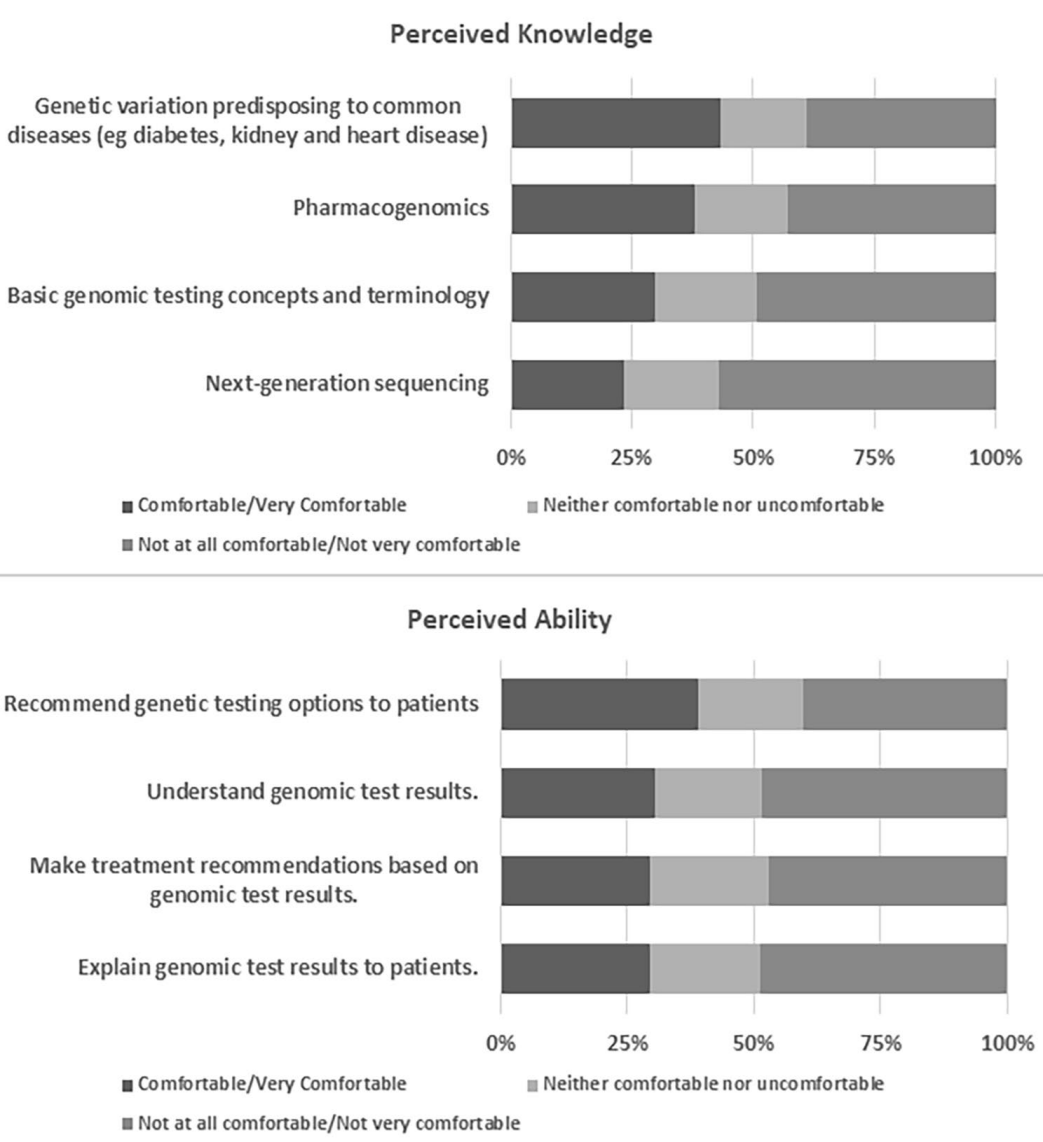
Distribution of knowledge and ability responses of participants

Knowledge scores of the respondents ranged from 4 to 20, with a median knowledge score of 12 (IQR: 8–14.5). Respondents were more comfortable about their knowledge of genetic variations predisposing to common diseases (43.3%, n = 130) and pharmacogenomics (38.0%, n = 114). They were least comfortable about their understanding of basic genomic testing concepts and terminology (29.7%, n = 89) and next-generation sequencing (23.3%, n = 70). The distribution of responses to knowledge questions is shown in Fig. 1.

On univariate analyses, respondents’ medical school year was significantly associated with their knowledge score (F [2,297] = 3.23, p = 0.04). Compared to those in their 4th year, students in their 6th year had a 1.54-point lower mean knowledge score (95%CI: -2.83, -0.24; p = 0.02) while those in 5th year had a 0.39-point lower mean knowledge score but this was not statistically significant (95%CI: -1.69, 0.92; p = 0.56). Students who indicated an interest in a career involving research had a borderline significant 1.03-point higher mean knowledge score compared to those who did not (95%CI: -0.03, 2.08; p = 0.06). Age, gender and ethnicity of participants did not show any significant associations with knowledge score of the participants.

After sequentially adjusting for age, gender, and interest in a research career, participants’ medical school year was significantly associated with knowledge score (F [2, 294] = 4.78, p = 0.009). Students in their 6th year had a statistically significant 2.16-point lower mean knowledge score than those in their 4th year (95%CI: -3.60, -0.72; p = 0.003). After adjusting for age, gender, and interest in a career involving research, each unit increase in medical school year was associated with a statistically significant 1.10-point lower mean knowledge score (F [1,295] = 8.97, p_trend_ = 0.003) [Table 2].

**Table 2:** Results of multivariate regression on knowledge score of respondents (N = 300)

	Model 1	Model 2	Model 3	Model 4	*P*_trend_
	beta (se)	beta (se)	beta (se)	beta (se)	
**Age**	0.06 (0.09)	0.03 (0.10)	0.19 (0.11)	0.18 (0.11)	
**Male**		0.61 (0.53)	0.63 (0.53)	0.64 (0.53)	
**5th year**			-0.66 (0.67)	-0.66 (0.67)	
**6th year**			-2.25 (0.73)^**^	-2.16 (0.73)^**^	0.003^**^
**Interested in a career involving research**				0.89 (0.53)	
**Adjusted** ***R*** ^**2**^	-0.00	-0.00	0.03	0.03	

^*^p < 0.05, ^**^p < 0.01, se = standard error, *P*_*trend*_*=* P value for trend

### Participants’ perceptions about their ability to apply genomics to clinical care

The ability scores of the respondents ranged from 4 to 20, with a median score of 11 (IQR: 7–15). Respondents were more comfortable about their ability to recommend genetic testing options to patients (39.0%, n = 117), to a lesser extent, understand genomic test results (30.3%, n = 91 and were least comfortable in their ability to make treatment recommendations based on genomic test results (29.3%, n = 88) and explain genomic test results to patients (29.3%, n = 88). The distribution of responses to ability questions is shown in Fig. 1.

On univariate analyses, respondents’ medical school year was significantly associated with ability scores (F [2,297] = 6.26, p = 0.002). Compared to students in their 4th year, students in their 5th year had a statistically significant 1.47-point lower mean ability score (95%CI: -2.84, -0.09; p = 0. 04) while students in their 6th year had a statistically significant 2.44-point lower mean ability score (95%CI: -3.81, -1.08; p < 0.001). In addition, each unit increase in knowledge score was significantly associated with a 0.77-point increase in mean ability score (95%CI: 0.69, 0.86; p < 0.001). Age, gender, ethnicity of participants and interest in a career involving research did not show any significant associations.

After multivariate adjustments for age, gender, medical school year, interest in a career involving research and knowledge score, participants’ knowledge score (β: 0.76 95%CI: 0.67, 0.84; p < 0.001), and medical school year (F [2,293] = 4.67, p = 0.01) were independent predictors of ability score. Compared to students in their 4th year, students in their 5th year had a 1.24-point lower mean ability score (95%CI: -2.21, -0.27; p = 0.01), and those in their 6th year had a 1.58-point lower mean ability score (95%CI: -2.66, -0.50; p = 0.004). After adjusting for age, gender, interest in a career involving research and knowledge score, each unit increase in medical school year was associated with a significant 0.78-point lower mean ability score (F [1,294] = 8.06, p_trend_ = 0.005) [Table 3].

**Table 3:** Results of multivariate regression on ability score of respondents (N = 300)

	Model 1	Model 2	Model 3	Model 4	Model 5	*P*_trend_
	beta (se)	beta (se)	beta (se)	beta (se)	beta (se)	
**Age**	0.04 (0.10)	0.04 (0.10)	0.25 (0.11)^*^	0.24 (0.11)^*^	0.11 (0.08)	
**Male**		0.13 (0.57)	0.22 (0.56)	0.22 (0.56)	-0.26 (0.39)	
**5th year**			-1.74 (0.70)^*^	-1.74 (0.71)^*^	-1.24 (0.49)^*^	
**6th year**			-3.26 (0.77)^***^	-3.22 (0.77)^***^	-1.58 (0.55)^**^	0.005^**^
**Interested in a career involving research**				0.49 (0.56)	-0.19 (0.39)	
**Knowledge Score**					0.76 (0.04)^***^	
**Adjusted** ***R*** ^**2**^	-0.00	-0.01	0.05	0.04	0.54	

^*^p < 0.05, ^**^p < 0.01, ^***^p < 0.001, se = standard error, *P*_*trend*_*=* P value for trend

### Attitudes of participants towards genome-guided prescribing and precision medicine

**Fig. 2:**
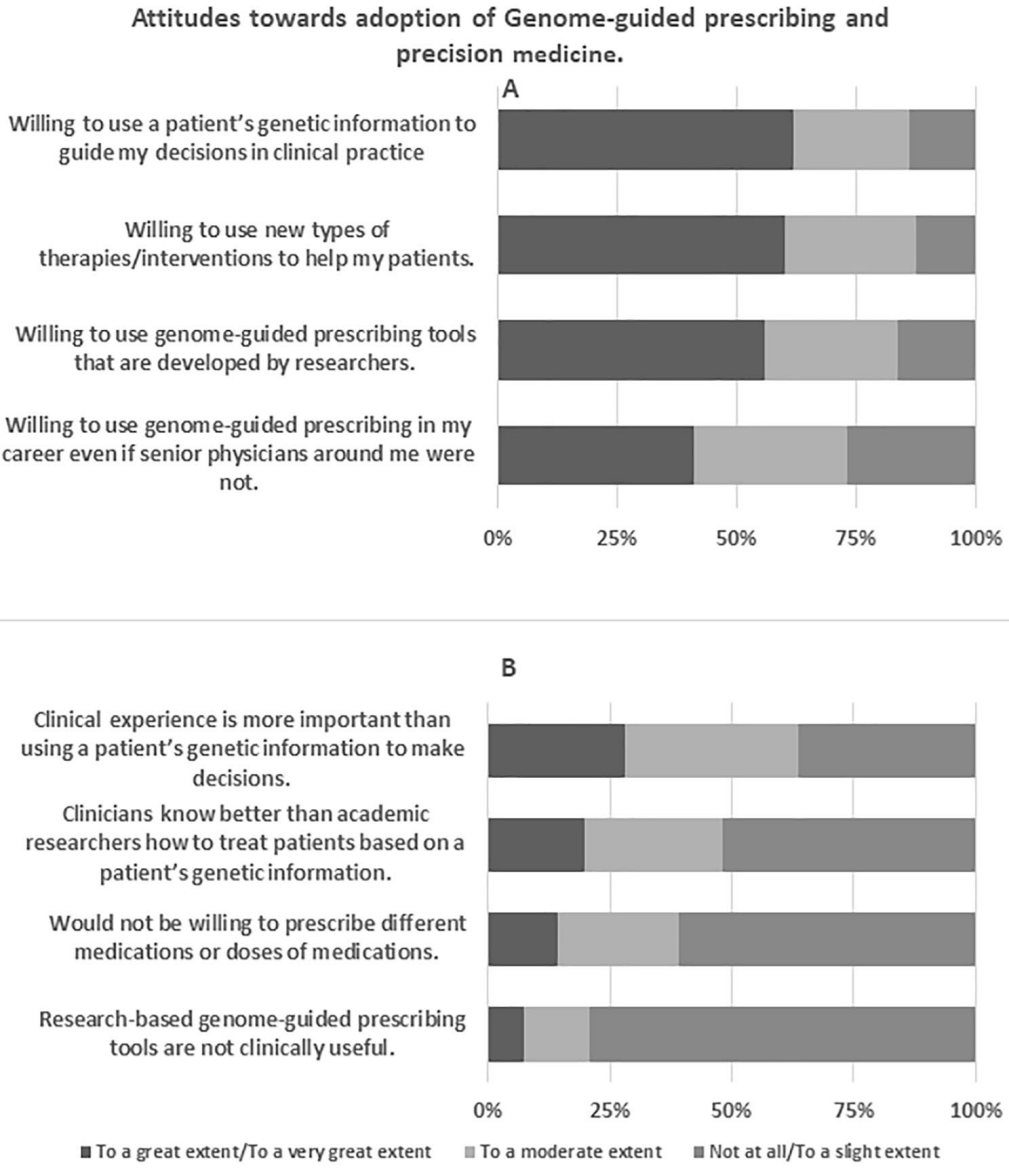
Distribution of participants’ responses to attitudes questions Respondents’ responses to questions assessing their attitudes towards the adoption of genome-guided prescribing and precision medicine. Section A includes the distribution of responses to openness questions while section B includes the distribution of responses to divergence questions

The attitude scores of participants ranged from 14 to 40, with a median score of 28 (IQR: 24–33). The median score on the openness items was 15 (IQR: 12–16). Respondents were more willing to use a patient’s genetic information to guide decisions in clinical practice (62.0%, n = 186), use new types of therapies to help patients (60.0%, n = 180), and use genome-guided tools developed by researchers (56.0%, n-168) but were less willing to use genome-guided prescribing in their career when senior physicians were not (41.0%, n = 123). The median score on the divergence items was 15 (IQR: 12–17). Respondents agreed that research-based genome-guided interventions were clinically useful (79.0%, n = 237), were willing to prescribe different medications or doses of drugs (61.0%, n = 183), to a lesser extent disagreed that clinicians know how to treat patients based on their genetic information better than researchers (52.0%, n = 156), and to a much lesser extent disagreed that clinical experience is more important than using a patient’s genetic information to make decisions (36.3%, n = 109). The distribution of responses to attitude questions is shown in Fig. 2.

On univariate analyses, each unit increase in knowledge score of the participants was significantly associated with a 0.14 decrease in mean attitude score (95%CI: -0.26, -0.02; p = 0.03). Age, gender, ethnicity, medical school year and interest in a career involving research were not significantly associated with attitude scores. Although the association with knowledge score persisted after adjusting for age and gender, adjusting for medical school year and interest in a career involving research resulted in a trend towards a null association. After maximal adjustment for age, gender, knowledge score, and interest in a research career, students in their 6th year had a significant 1.65-point higher mean attitude score than those in their 4th year (95%CI: 0.75, 3.23; p = 0.04). However, medical school year overall was not significantly associated with attitude scores (F [2,293] = 2.50, p = 0.08). Nevertheless, after maximal adjustment, each unit increase in medical school year was significantly associated with a 0.81-point increase in mean attitude scores (95%CI: 0.02, 1.60; p_trend_ = 0.04) [Table 4]. Likelihood ratio chi-square tests did not reveal any evidence of statistical interaction between knowledge scores and medical school year (X^2^ = 2.66, p = 0.26).

**Table 4:** Results of multivariate regression on attitude score of respondents (N = 300)

	Model 1	Model 2	Model 3	Model 4	*P*_trend_
	beta (se)	beta (se)	beta (se)	beta (se)	
**Knowledge Score**	-0.14 (0.06)^*^	-0.13 (0.06)^*^	-0.11 (0.06)	-0.12 (0.06)	
**Age**		-0.10 (0.10)	-0.18 (0.12)	-0.19 (0.12)	
**Male**		-0.60 (0.57)	-0.69 (0.57)	-0.67 (0.57)	
**5th year**			1.40 (0.73)	1.39 (0.72)	0.04^*^
**6th year**			1.57 (0.80)	1.65 (0.80)^*^	
**Interested in a career involving research**				1.09 (0.57)	
**Adjusted** ***R*** ^**2**^	0.01	0.01	0.02	0.03	

^*^p < 0.05, se = standard error, *P*_*trend*_*=* P value for trend

### Ethical concerns related to precision medicine

**Fig. 3:**
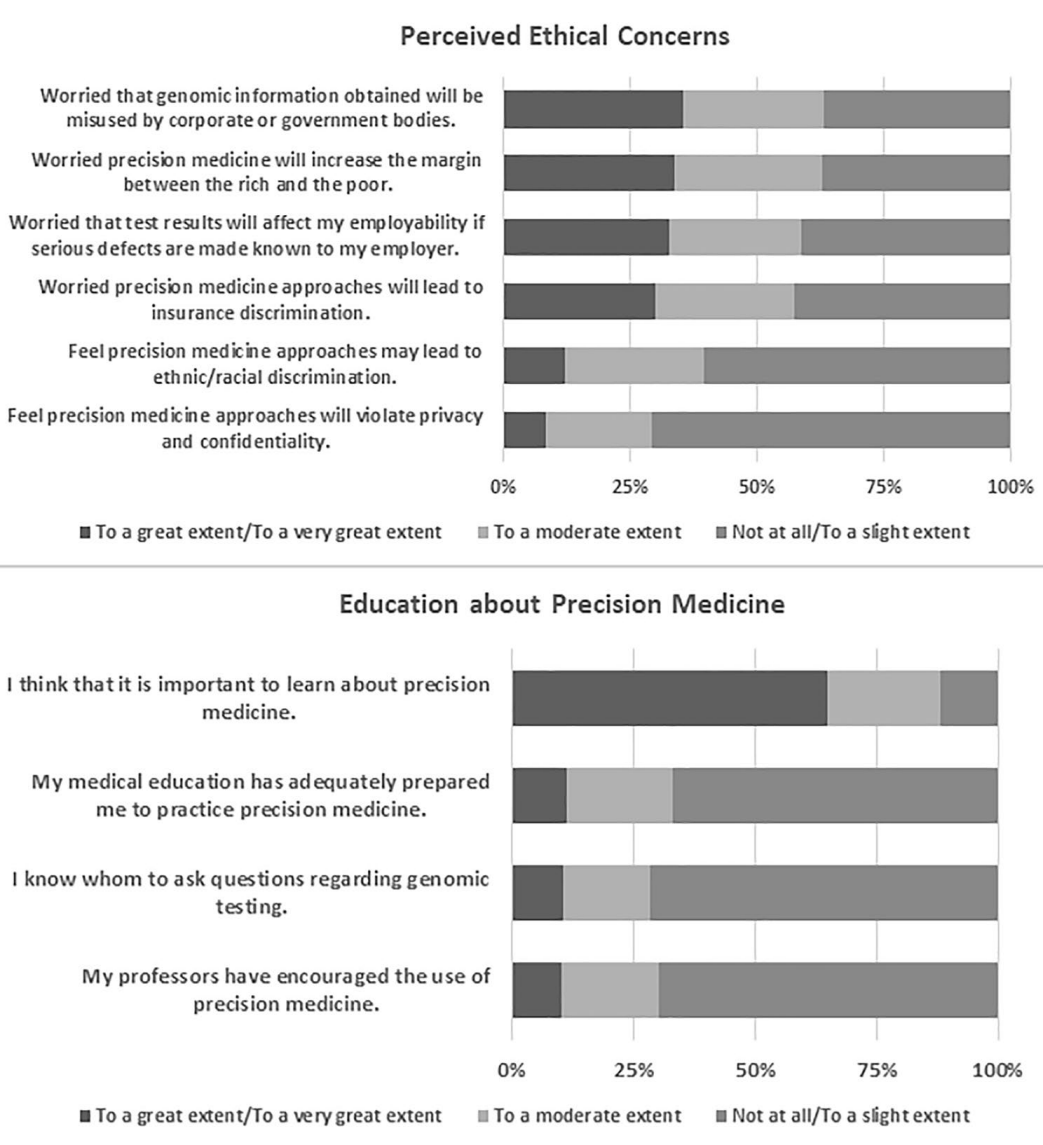
Respondents’ perceptions of ethical concerns and education about Precision Medicine

The distribution of ethical concerns expressed by respondents is shown in Fig. 3. More than a quarter of the respondents were worried that genomic information obtained would be misused by government and corporate bodies (35.7%, n = 107) and that their application would increase margins between the rich and the poor (34.0%, n = 102). A similar proportion were worried that results from tests can affect employability if serious genetic defects are made known to their employers (33.0%, n = 99) and that they will lead to insurance discrimination (30.0%, n = 90). However, less than a quarter of the respondents felt that precision medicine approaches would lead to ethnic/racial discrimination (12.3%, n = 37), and only 8.7% (n = 26) of the respondents felt that precision medicine approaches would violate privacy and confidentiality.

### Perceptions about education in precision medicine

Most respondents (65.0%, n = 195) thought it was important to learn about precision medicine. Only 11.3% (n = 34) of the respondents felt that their education had adequately prepared them to practice precision medicine. Only 10.7% (n = 32) thought they knew who to ask about genomic testing. Finally, only 10.3% (n = 31) of the respondents felt their professors had encouraged the use of precision medicine. The distribution of responses to education items is shown in Fig. 3.

## Discussion

Our study assessed the awareness, perceptions about knowledge, ability and attitudes to adopt precision medicine approaches in the clinical management of patients as well as perceptions of ethical concerns and their education in precision medicine among 300 medicine and surgery students in clinical years of two Nigerian medical schools. The study sample included a reasonably equal representation from all levels, with the lowest being 4th-year students (25.3%) and most students expressing interest in a career involving research (63.3%).

Although our findings showed a high level of awareness (92.6%) of at least one of the precision medicine terminologies surveyed, the knowledge items notably revealed lowest knowledge of basic genomics testing concepts and terminology (29.7%) and next-generation sequencing (23.3%). Overall, less than half of the respondents indicated they were at least comfortable with their knowledge of each of the four knowledge items tested. Our findings are similar to those from a recent review where only 28% of medical and pharmacy students had adequate knowledge of pharmacogenomics [15]. However, unlike the studies reviewed, our study found no significant differences in knowledge of students with respect to age and gender. Nevertheless, the level of awareness and knowledge in this study was higher than that reported in a recent survey of health students in Saudi-Arabia [13].

Interestingly, after maximal adjustments, the knowledge score was significantly higher among 4th-year students than among 6th-year students, and the knowledge score significantly decreased with increasing medical school year.

In addition, less than half of the respondents reported being comfortable with their ability to apply genomics to clinical care across all ability items. However, after maximal adjustments, our study found significantly higher ability scores among 4th-year students than among 5th and 6th-year students, with a significant decrease in ability scores with increasing medical school year. Also, higher knowledge score was independently associated with higher ability scores after maximal adjustments.

On the contrary, Eden et al. (2016) reported significantly lower knowledge and ability in the first year students compared to older years, while Bakry et al. (2022) found no differences in the knowledge of the respondents by medical school year [12, 13]. The trend toward lower knowledge and ability scores from 4th years who had recently completed basic medical science training to more senior years who were completing clinical training may be an effect of a recent curriculum change in the University of Lagos, affecting students in the 4th year class and below. However, this trend highlights significant training needs for students in their clinical years to sustain and improve their knowledge of genomic medicine concepts and the integration of these concepts in clinical practice.

Regardless, students in our study had overall positive attitudes and expressed openness to precision medicine and genome-guided prescribing even when they diverged from usual care. The overall positive attitudes in this survey are consistent with previous surveys in other regions even when level of knowledge was reportedly low [12, 13, 15]. Although knowledge scores appeared to show significant inverse associations with attitudes even after adjustments for age and gender, additional adjustment for medical school year resulted in a trend towards the null. This suggests that medical school year likely confounded or mediated the inverse association between knowledge scores and attitude scores. The significant trend towards higher attitude scores among 5th and 6th years than among 4th years suggest stronger favorable dispositions towards precision medicine among older clinical students regardless of their knowledge.

Furthermore, our findings highlighted notable ethical concerns regarding precision medicine among our respondents, most of which bothered around the use of genomic testing results by governments or corporate bodies, possible widening of socioeconomic disparities, employability and insurance discrimination. Interestingly, respondents were least concerned about violation of privacy and confidentiality and ethnic/racial discrimination. These concerns closely reflected similar findings by Siamoglou et al. (2021) and Mahmutovic et al. (2018) [1, 14]. In contrast, however, in Mahmutovic et al. (2018), students were most concerned about privacy and confidentiality. With the advances in genomic medicine being championed by private corporate entities in Nigeria and Africa, this finding highlights a need to safeguard trust and trustworthiness in informed consent and the subsequent use and dissemination of participants’ genomic data by corporate bodies by ensuring the highest ethical standards are maintained [31]. In addition, issues surrounding the effects of adopting precision medicine approaches on health and socioeconomic disparities and discrimination based on genetic data have been discussed and are likely to be worse in low-income countries with already existing disparities [32, 33]. It is, therefore, crucial that strategies anticipating these issues should be taken into account as countries continue to leverage precision medicine.

Finally, our study highlights significant gaps in students’ perceptions about their education in precision medicine. Similar to the findings of Mahmutovic et al. (2018) and Eden et al. (2016) [1, 12], the majority of the respondents in our study (65.0%) felt it was important to learn about precision medicine. Nevertheless, most respondents felt their education had not prepared them for precision medicine, their professors had not encouraged precision medicine approaches, and most felt they did not know whom to ask about genetic testing. Similarly, Eden et al. (2016) in the United States showed only a minority of students agreed with these statements [12]. In contrast, 51% of medical students in Bosnia and Herzegovina felt their education was well-designed for precision medicine [1]. This is the first study evaluating medical students’ perception of their precision medicine education in Nigeria. Afolaranmi et al. (2021) [27] highlighted the poor state of genomics education in Nigeria, including the lack of sufficient quality laboratory exposure for medical students, resulting in a consequent relative lack of genomics-based research. Our findings support the need for an improved nationwide medical school curriculum in Nigeria that is congruent with the current practice needs of modern medicine. This will update the interests and expertise of future Nigerian physicians to make them competitive in the global medical research space.

The strengths of our study lie in its being the first to assess the perception of a fairly large cross-section of Nigerian medical students from two medical schools in Nigeria’s most populous state regarding their perceptions about their knowledge and ability, attitudes, ethical concerns and education about precision medicine, to highlight training and policy needs. However, our study had a few limitations. First, the reported items were based on students’ subjective perceptions, although we believe an objective assessment is more likely to confirm the gaps observed in our study. Also, regardless of existing efforts in medical education, if students’ perceptions about their education are still below expectations, then gaps still exist. Secondly, the cross-sectional design limits any causal inferences from this study as changes in perceptions of over time are not captured. In addition, there was a possibility of misclassification errors in the medical school year due to the nationwide university strike action, which variably affected student transition into their next school years and may have constituted a difference in how respondents answered this item. Also, minor differences in medical curriculum between both schools may have caused a variation in knowledge experienced among the various years, but this would have biased our findings towards the null. Furthermore, as this study surveyed only medicine and surgery students from two medical schools in Lagos, Nigeria, findings may not be generalizable to students from other departments or medical schools. The non-random sampling methodology employed may additionally subject the findings to selection bias. Finally, the relatively wider confidence intervals in some sub-levels and nominally significant results suggest the possibility of chance findings. As such, this necessitates larger, more representative nationwide prospective studies to fully assess preparedness for precision medicine among medical students in Nigeria.

## Conclusion

Our study has highlighted a high awareness of precision medicine terminologies among medicine and surgery students in Lagos, significant gaps in medical students’ perceptions of their knowledge of precision medicine concepts and ability to apply genomic medicine in clinical practice, an overall positive attitude towards the adoption of precision medicine, notable ethical concerns about genomic medicine applications as well as poor perceptions about their education regarding precision medicine. Our findings call for improved training of medical students in precision medicine to prepare them for this fast-growing approach, more robust research assessing the state of genomics education in Nigeria, and careful attention to the ethical concerns surrounding the collection, use and dissemination of genomic test results.

### Electronic supplementary material

Below is the link to the electronic supplementary material.

Supplementary Material 1

## Data Availability

The datasets used and analysed during the current study are available from the corresponding author on reasonable request.

## Abbreviations

H3Africa: Human Heredity and Health in Africa
NBGN: Nigerian Bioinformatics and Genomics Network
MDCN: Medical and Dental Council of Nigeria
EBPAS: GII-Evidence-based Practice Attitude Scale Adapting Genome-informed Interventions
